# Molecular clustering and prognostic features based on integrated databases predict survival and immune status in patients with gastric cancer

**DOI:** 10.3389/fonc.2025.1642911

**Published:** 2025-09-04

**Authors:** Yin Shi, Jiaying Zhou, Keping Jia, Hao Song, Tianlong Zhang, Weiwei Yuan, Jiahao Ge

**Affiliations:** ^1^ Department of Hepatobiliary and Pancreatic Surgery, Jinhua Hospital Affiliated to Zhejiang University, Jinhua, Zhejiang, China; ^2^ Department of Internal Medicine, Yiwu Maternity And Children Hospital, Yiwu, Zhejiang, China; ^3^ Department of Gastroenterology, the Fourth Affiliated Hospital of School of Medicine, and International School of Medicine, International Institutes of Medicine, Zhejiang University, Yiwu, China; ^4^ Department of Traditional Chinese Medicine, Yiwu Maternity And Children Hospital, Yiwu, Zhejiang, China; ^5^ Department of General Surgery, The First Affiliated Hospital of Anhui Medical University, Anhui Public Health Clinical Center, Hefei, China; ^6^ Department of Critical Care Medicine, the Fourth Affiliated Hospital of School of Medicine, and International School of Medicine, International Institutes of Medicine, Zhejiang University, Yiwu, China; ^7^ Department of Thyroid Surgery, Baoshan Hospital Affiliated to Shanghai University of Traditional Chinese Medicine, Shanghai, China

**Keywords:** gastric cancer, molecular clustering, prognostic features, immune microenvironment, biomarkers

## Abstract

**Background:**

Gastric cancer (GC) remains one of the most common malignancies worldwide with high mortality rates despite advances in treatment approaches. Patients frequently develop drug resistance to current therapies, highlighting the critical need for novel prognostic biomarkers that can enhance survival rates and guide immunotherapy decisions in patients with GC.

**Methods:**

We conducted a comprehensive bioinformatics analysis using integrated clinical data from The Cancer Genome Atlas (TCGA) and Gene Expression Omnibus (GEO) databases. GC cases were categorized into two prognostic-related gene (PRG) clusters, and differentially expressed genes were identified. We established a prognostic model based on 11 key genes, stratified patients into high-risk and low-risk groups, and developed a nomogram model for survival prediction. Expression of selected genes was validated through quantitative real-time polymerase chain reaction (qRT-PCR) and immunohistochemistry in clinical samples.

**Results:**

The identified PRGs and gene clusters strongly associated with patient survival, immune system functions, and cancer-related pathways. Risk scores significantly correlated with immune cell abundance, checkpoint expression, and responses to immunotherapy and chemotherapy. For instance, the area under the curve (AUC) values of patients at 1-year, 3-year, and 5-year survival were all greater than 0.6 in the ROC curves (*p* < 0.05), which makes our prediction more accurate, and the line graphs predicted a 1-year survival rate exceeding 0.907, a 3-year survival rate exceeding 0.726, and a 5-year survival rate exceeding 0.633; the calibration curves are almost close to the predicted ones (*p* < 0.05). This implies that patients in the high-risk group demonstrated significantly poorer prognosis. Univariate Cox (UniCox) analysis and multivariate Cox (MultiCox) analysis indicate that CTHRC1 (Collagen Triple Helix Repeat Containing 1), CST6 (Cystatin E/M), and AKR1B1 (Aldo-Keto Reductase Family 1 Member B) are independent prognostic factors, and all are associated with poor survival prognosis (HR > 1, *p* < 0.05). Gene set enrichment analysis (GSEA) and single-cell analysis revealed significant enrichment of multiple biological pathways and variability in expression of these genes across different cell types within the tumor microenvironment. qRT-PCR and immunohistochemistry confirmed significant differences in mRNA and protein expression of CTHRC1, CST6, and AKR1B1 between normal and GC tissues (*p* < 0.05).

**Conclusion:**

Our research establishes a robust molecular signature for predicting survival of patients with GC and characterizing the tumor immune microenvironment. It aims not only to establish a prognostic model, but also to explore immunobiological functions. The identified prognostic features and key genes (CTHRC1, CST6, and AKR1B1) offer potential as biomarkers and therapeutic targets, potentially guiding more effective personalized treatment strategies for patients with GC.

## Introduction

Gastric cancer (GC) is a major global health challenge, ranking as the fifth most commonly diagnosed cancer and the fourth-leading cause of cancer-related deaths worldwide (1). Each year, approximately 1 million individuals worldwide are diagnosed with GC, leading to an estimated 738,000 deaths (2). The disease exhibits remarkable molecular and phenotypic heterogeneity, with risk factors including *Helicobacter pylori* infection, advanced age, high salt intake, and a diet low in fruits and vegetables (3). Despite advancements in surgical techniques, radiotherapy, and immunotherapy over the past decade, the mortality rate associated with GC remains alarmingly high (4). The pathogenesis of GC involves genetic mutations, chromosomal abnormalities, differential gene expression, and epigenetic modifications. The tumor microenvironment (TME) facilitates immune evasion, which contributes to resistance against conventional and immunotherapeutic treatments (5, 6). Therefore, a deeper understanding of the molecular characteristics of GC and the role of immunosuppressive systems within this specific environment is imperative (7). This not only aids in elucidating the mechanisms of GC progression but also is crucial for developing innovative treatment strategies.

As high-throughput sequencing technologies continue to advance, researchers now have access to a wealth of sequencing data from public databases such as The Cancer Genome Atlas (TCGA) and the Gene Expression Omnibus (GEO). In recent years, numerous studies have focused on leveraging these data to construct tumor classifications or prognostic features, aiming to predict survival and immune status in various malignancies. For instance, Yao et al. developed techniques to identify the immune infiltration microenvironment in cervical cancer and constructed an immune scoring system, analyzing their correlations with patient prognosis and immune therapy responses (8). These prognostic indicators are valuable for predicting the survival rates and immune characteristics of patients with cervical cancer, as well as their potential benefits from immune checkpoint inhibitor (ICI) therapy. Wang et al. utilized hepcidin antimicrobial peptide (HAMP) to identify pivotal genes associated with HAMP and developed a prognostic prediction model. They explored the synergistic effects of HAMP with immune cells and chemokines, and its potential role in inhibiting the progression of gallbladder cancer (CHOL) (9). Furthermore, a previous study using TCGA data identified six immune subtypes, covering all types of human malignancies, namely, wound healing, IFN-γ dominant, inflammatory, lymphocyte-depleted, immune quiet, and transforming growth factor-β (TGF-β) dominant. These subtypes are closely associated with patient prognosis, as well as genetic and immune features. Additionally, genes related to cell death and long non-coding RNAs have also been employed to construct tumor classifications and prognostic features, further expanding the boundaries of cancer biology research.

Although many of the algorithms used in machine learning were developed decades ago, the emergence of big data and significant advancements in computational power over the past 20 years have rekindled interest and broadened the application of this technology (10). This is particularly evident in the field of medicine, where the potential of machine learning is being fully exploited, especially in oncological pathology. Machine learning algorithms are capable of processing complex datasets and performing crucial tasks such as tumor diagnosis, subtyping, pathological grading, clinical staging, and prognosis prediction (11). Moreover, these algorithms can effectively identify pathological features, biomarkers, and genetic variations associated with tumors, which are vital for developing personalized treatment strategies. By analyzing pathological images and genetic information in depth, machine learning not only enhances the accuracy of diagnoses but also accelerates the optimization of treatment protocols and the development of new drugs (12). The further advancement of this technology is expected to revolutionize traditional pathological methods, making them more automated, precise, and efficient.

Our research demonstrates that molecular clustering and prognostic features derived from combined data from the TCGA and GEO databases can predict the prognosis and intratumoral immune landscape of patients with GC. Initially, data downloaded from TCGA and GEO were merged and organized, and two discrete prognostic-related gene (PRG) clusters were constructed based on expression levels. Subsequently, patients were divided into two clusters based on differentially expressed genes (DEGs) identified from the two PRG clusters. Further calculations of risk scores were conducted and validated, and prognostic features were established to predict the overall survival (OS) and response to immunotherapy in patients with GC. Next, we used immunohistochemical (IHC) staining from the Human Protein Atlas (HPA) website to validate the three genes [CTHRC1 (Collagen Triple Helix Repeat Containing 1), CST6 (Cystatin E/M), and AKR1B1 (Aldo-Keto Reductase Family 1 Member B)] of prognostic models. Finally, we applied quantitative real-time polymerase chain reaction (q-PCR) to validate CTHRC1, CST6, and AKR1B1 expression levels in clinical GC tissue samples to verify our results. This signature not only serves as an independent prognostic marker for patients with GC and a predictor of clinical characteristics, but also significantly differentiates patients who are more sensitive to chemotherapeutic agents and immunotherapy.

## Materials and methods

### Downloading transcriptome data and acquiring clinical information

A total of 162 tumor tissues and 50 normal tissues of patients with GC were first sourced from transcriptomic data and gathered from GEO datasets (microarray platforms) (https://www.ncbi.nlm.nih.gov/geo/) (GSE65801 and GSE66229); subsequently, we downloaded clinical data of patients with GC and integrated the data. We translated the gene symbol ID to gene name in transcriptome data, and used Perl software (v5.30.0) and R software (v4.1.2) to collate the downloaded data. Sex, age, grading, and pathological TNM staging were obtained from the clinical data and integrated. Incomplete clinical information was removed.

### Acquisition of DEGs and visualization

DEGs of GC-related genes were found using the “limma” package of R software to visualize DEGs in normal and tumor samples in GC. The critical value standard for identifying DEGs was set as *p*-value < 0.05 and |logFC (fold change)| > 1. Heatmaps and volcano plots were constructed to visualize the expression of the DEGs.

### Protein–protein interaction network analysis

The STRING database (http://string-db.org) was utilized to construct the protein–protein interaction (PPI) network of DEGs. PPI visualization of the association of proteins encoded by differential GC-related genes (interaction score > 0.40) was limited to “*Homo sapiens*”. We utilized the Cytoscape software to import the data files for visual editing (https://cytoscape.org/).

### WGCNA analysis

Weighted gene correlation network analysis (WGCNA) with the expression profile using the R package “WGCNA” was used to construct the gene coexpression networks of TCGA-GC (tumor and normal samples). The process of network construction primarily involves the following steps: (1) Define the matrix. (2) Transform the matrix into a topological overlap matrix (TOM). (3) Layer the dissTOM based on Tom Cluster to acquire a hierarchical clustering tree. (4) A dynamic tree-cut method was applied to extract modules from the hierarchical clustering dendrogram. (5) The module eigengenes (MEs) were calculated for each module, which represent the overall expression level of the module. The Pearson correlation coefficient was calculated for the MEs of each module, and the average distance between the MEs of each module was defined as 1−Pearson correlation coefficient. We used the average linkage hierarchical clustering method to cluster the MEs of all modules, and the minimum value (genome) was set to 100. Next, using the PickSoft threshold function, we initially evaluated the scale-free topology fit index (*R* ^2^) and mean connectivity across a range of 1–20. At soft threshold = 9, *R* ^2^ stably exceeded 0.80 (approaching 0.84) for the first time, while the slope of the curve approached a plateau. This value represents an optimal trade-off between approximating scale-free topology and retaining sufficient network connectivity.

### The construction of the risk scoring system

We divided the GC data into two cohorts: a training cohort, which was employed to construct the prognostic model, and a test cohort, which was employed to validate the accuracy of the prognostic model values for GC-related genes. Risk score formula: risk score = ∑i1 (Coefi*ExpGenei). “Coefi”, regression coefficient; “ExpGenei”, gene expression. Samples were categorized into high- and low-risk groups based on the median value of risk score for each sample. By 10-fold cross-validation, prognosis-related false-positive GC-related genes were first eliminated by least absolute shrinkage and selection operator (LASSO) Cox regression analysis. We found that the characteristic genes of the model are the set of genes corresponding to the point with the smallest error. Then, the prognosis-related GC-associated genes were evaluated using a multifactorial Cox regression analysis model to analyze OS and clinical outcomes in patients with GC. Finally, we used the predicted independent prognostic gene sets to further construct the prognostic model. Between the two cohorts, we used the “survival” and “monitor” packages to plot Kaplan–Meier curves to analyze survival differences (including 5-year survival), and used the “timeROC” package to plotROC curves to predict the predictive accuracy of the two cohort characteristics.

### Evaluation and validation of prognostic models

We included the genes screened from WGCNA, input each gene individually, and compared it with survival time and survival status to screen out independent predictors of prognosis in patients with GC by univariate Cox (UniCox) regression analyses. *p* < 0.01 was regarded significant. Time-dependent ROC curves were utilized to assess the ability of the risk score to predict OS. The C-index was used to compare differences in the efficiency of risk scores and clinicopathological factors in predicting the prognosis of patients with GC. Factors with significant outcomes in multivariate analysis were used to construct the nomogram to facilitate individualized assessment of each case. We plotted calibration curves to assess whether the predicted patient (1-year, 3-year, and 5-year) survival probabilities of the nomogram were close to the true probabilities.

### Immunotherapy prediction and immunohistochemical staining

To evaluate the relationship between immune cell content and risk score, the immune cell infiltration data files for all TCGA tumor types were obtained from the TIMER 2.0 database (http://timer.cistrome.org/). ggplot2, ggtext R, Limma, and scales packages were used to generate bubble plots to reveal the correlation between immune cell content and risk scores. The official LM22 signature (v2023-06-15) was applied for CIBERSORT, with input data formatted as a log2(TPM+1) matrix. Quantile normalization was disabled (QN=FALSE), and permutation was set to 1,000. Only samples with *p* < 0.05 were retained for downstream analysis. xCell (v1.1.0), TIMER (v2.0), QUANTISEQ (v1.3), MCPCOUNTER (v1.2.0), and EPIC (v1.1.5) were run concurrently, gene set versions matched their respective software defaults, and a uniform threshold of *p* < 0.05 was applied for filtering. The “limma” R software package was used to analyze the difference in immune cell content between the high-risk group and low-risk group and generate a heatmap by utilizing the pheatmap R software package. We used immune cell difference analysis to evaluate the grade of tumor immune infiltration in different types. The differences in immune cells and immune function between the high-risk and low-risk groups were shown by the Wilcoxon signature rank test with boxplots. We calculated the immune and stromal scores by using the ESTIMATE algorithm in GC to predict the content of immune and stromal cells. In addition, we used Wilcoxon signed rank test to compare the immune checkpoint expression between the high-risk and low-risk groups. Analyses were performed using the TIDE web platform (v2.0) with default parameters, with internal TPM-scale normalization. Responder probability thresholds were set at the officially recommended cutoff of 0.25. We obtained IHC staining images of different proteins CTHRC1, CST6, and AKR1B1 in GC and normal tissues from the HPA website (https://www.proteinatlas.org/).

### Gene set enrichment analysis

We stratified samples into high-expression and low-expression groups based on CTHRC1, CST6, and AKR1B1 expression levels first. Specifically, the top 30% of samples with the highest CTHRC1, CST6, and AKR1B1 expression were designated as the high-expression group, while the bottom 30% were assigned to the low-expression group. To identify DEGs between the two groups, differential expression analysis was conducted using “limma” in the R package. During this analysis, we calculated log2 fold change (log2FC). Following DEG analysis, genes were ranked based on log2FC, and the ranked gene list enabled a clearer representation of expression trends and served as the input for gene set enrichment analysis (GSEA); GSEA was performed using the GSEA function from the “clusterProfiler” package, leveraging multiple gene set databases including GO Biological Process (GO-BP), GO Molecular Function (GO-MF), GO Cellular Component (GO-CC), Reactome, and WikiPathways. The analysis computed the normalized enrichment score (NES) for each gene set and assessed statistical significance using both permutation testing and multiple hypothesis correction. *p* < 0.05 was considered statistically significant.

### The clinical correlation analysis of genes CTHRC1, CST6, and AKR1B1

In this study, we performed UniCox and multivariate Cox (MultiCox) proportional hazards regression analyses to evaluate the hazard ratio (HR) for predicting the clinical relevance of these genes. We used the “survival” package in R to evaluate the association between gene expression and clinical variables. For each variable, the HR and the corresponding 95% confidence interval (CI) were calculated to quantify relative risk. To facilitate visualization, forest plots were generated using the “forestplot” package, illustrating effect sizes along with their CIs.

### Single-cell analysis

In this study, single-cell RNA sequencing data from the GEO datasets EMTAB8107 and GSE167297 were used to analyze CTHRC1, CST6, and AKR1B1 expression across stomach adenocarcinoma (STAD) tissues. After analyzing the data by using the Seurat R package, uniform manifold approximation and projection (UMAP) was applied to visualize CTHRC1, CST6, and AKR1B1 expression in various cell clusters, with focus on cells such as CD8^+^ T, epithelial, and pit mucous cells. AUCell was used to score biological pathways in CTHRC1-, CST6-, and AKR1B1-expressing cells, specifically proliferation-related pathways. Spearman’s correlation analysis further revealed the relationship between CTHRC1, CST6, and AKR1B1 expression and pathway activity.

### Quantitative real-time PCR

Paired cancer tissue samples from nine patients with GC were collected from the Department of General Surgery, The First Affiliated Hospital of Anhui Medical University. Informed consent was obtained from all participants or their authorized representatives, and the study design complied with the ethical standards stipulated by the institutional review board. All patients had no previous immune-related diseases and no preoperative neoadjuvant chemotherapy. TRIzol reagent was employed to isolate total RNA from the tissue samples, which was subsequently reverse transcribed into cDNA for quantitative real-time polymerase chain reaction (qRT-PCR). Following the activation of the CT value for the target sample, the relative expression level of the target gene was assessed by 2^−ΔΔCt^ with the adjacent tissue as the control. Human GAPDH was used as an internal reference. The differential expression of three pairs of GC-related genes between GC tissues and adjacent non-cancerous tissues were assessed by using *t*-test. Graphs were generated by using the GraphPad Prism 8.0 software. The primer sequences used in this study are listed in **Supplementary Table S1**.

### Statistical data analysis

We analyzed the data by using R 4.1.0 software and used Strawberry Perl-5.32.1.1 to run the script in the script analysis, followed by analyzing normal distribution by using Student’s *t*-test and non-normal distribution parameters by using Wilcoxon rank sum test. Pearson chi-square test was used to analyze statistical data. *p < 0.05, **p < 0.01, and ***p < 0.001 were considered statistically significant.

## Results

### Objectives and study workflow

**Figure 1:**
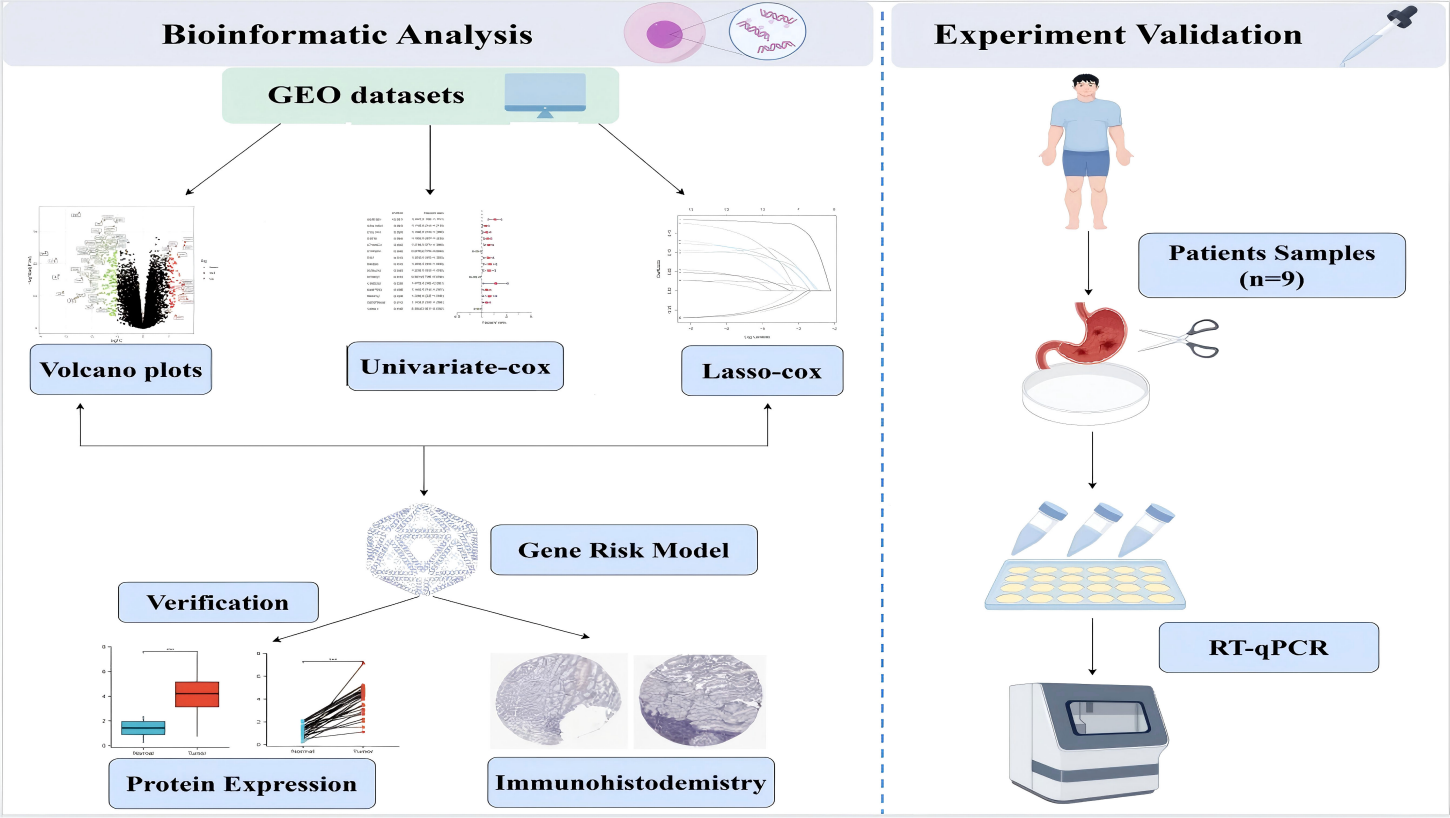
The flowchart of this study.

This study aims to demonstrate that molecular clustering and prognostic features derived from integrated data from the TCGA and GEO databases can effectively predict the prognosis and intratumoral immune landscape of patients with GC for elucidating the mechanisms of GC progression and developing innovative treatment strategies. We used a comprehensive bioinformatics methodology, functional analysis, and some experimental validation; the flowchart of this study is shown in **Figure 1**.

### Identification of differentially expressed genes in GC

**Figure 2:**
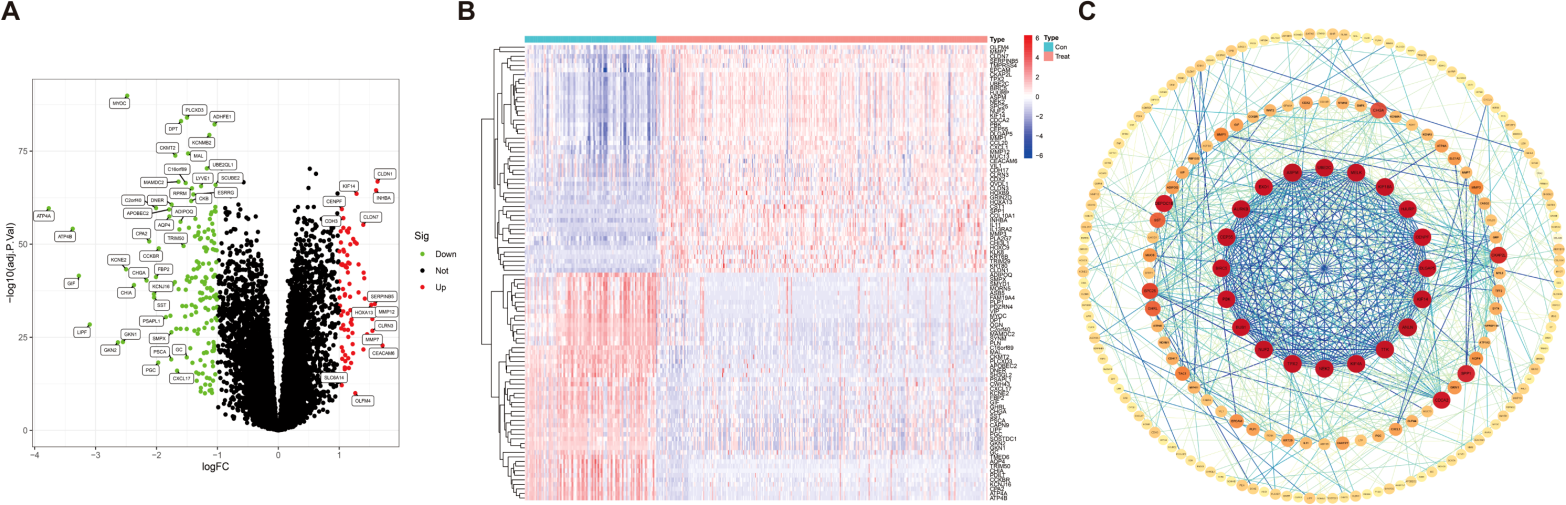
Identification of differentially expressed genes in gastric cancer. **(A)** Volcano map of 263 differentially expressed genes [|logFC (fold change)| > 1, *p* < 0.05]. **(B)** Heatmap for differentially expressed genes in GC identified using the GEO database (microarray platforms). **(C)** Network circle diagram depicting protein–protein interactions among GC-associated genes.

In the GEO database, we obtained gene expression data for a cohort of 162 GC samples from GSE65801 and GSE66229. By comparing the expression patterns across GC samples and normal samples (logFCfilter = 1, fdrFilter < 0.05), we successfully identified 263 DEGs (**Supplementary Table S2**), as shown in volcano plots (**Figure 2A**) and heatmaps (**Figure 2B**). The horizontal axis represents Log2 (fold change), with points farther from the center indicating a greater fold difference. The vertical axis represents −Log10 (adjusted *p*-value), where points positioned higher on the axis correspond to a more statistically significant difference. Among these, the red dots represent upregulated genes, and the green dots represent downregulated genes. In comparison to the upregulated genes marked in red, the downregulated genes represented in green exhibit more significant differences and greater fold differences. To identify DEG-associated interacting proteins, we input 263 DEGs into the STRING website, then constructed gene interaction networks and PPI networks potentially associated with GC-related genes. Data files were imported into Cytoscape software for visual editing. The core functional modules of the PPI network consist of 20 genes, namely, UBE2C, MELK, KIF18A, HJURP, CENPF, DLGAP5, KIF14, ANLN, TTK, KIF4A, NEK2, TPX2, NUF2, BUB1, PBK, BIRC5, CEP55, AURKB, EXO1, and ASPM (**Figure 2C**).

### Identification of key pivotal hub genes via weighted gene correlation network analysis

**Figure 3:**
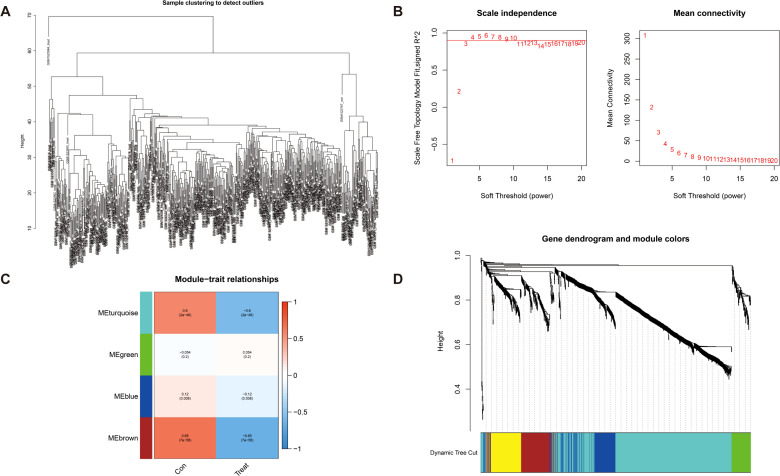
Detection of hub genes in gastric cancer through the application of the WGCNA framework. **(A)** Clustering analysis for the identification of outlier samples. **(B)** The soft threshold is ascertained by function. The left panel illustrates the correlation between the scale-free topology fit index (*R*²) and the soft threshold, and the right panel depicts the association between mean connectivity and the soft threshold. **(C)** Correlation analysis between merged modules and clinical traits, correlation coefficients, and *p*-values presented within the respective color modules. **(D)** Hierarchical clustering of genes was visualized as a dendrogram through the aggregation of homologous modules.

DEGs were screened using the “DESeq2/edgeRpackage” software from the GEO database (logFCfilter = 1, fdrFilter < 0.05). Subsequently, WGCNA (R package v1.72) was employed to construct a genome-wide co-expression network for all DEGs (the minimum module genome value was set to 100), identifying clinical trait-associated modules (soft threshold = 9, *R* ^2^ = 0.84), to explore key genes with biological significance in GC samples. As shown in **Figure 3B**, the optimal soft threshold was set to 9 (goodness of fit *R^2^* = 0.84; **Supplementary Figure S1**). Gene clustering was performed using a predefined threshold, ensuring a minimum of 60 genes per module, which ultimately resulted in four distinct modules (**Figures 3A, C, D**). Next, we computed the correlation coefficients between each gene module and the samples derived from both training set and test set of DEGs. Through a meticulous evaluation of correlation coefficients and related *p*-values, we identified a significant association between the brown module and clinical traits, with a correlation coefficient of 0.65 and *p*-value of <0.001 (**Figure 3C**, **Supplementary Figure S2**).

### Construction and evaluation of prognostic characterization of DEGs in GC

**Figure 4:**
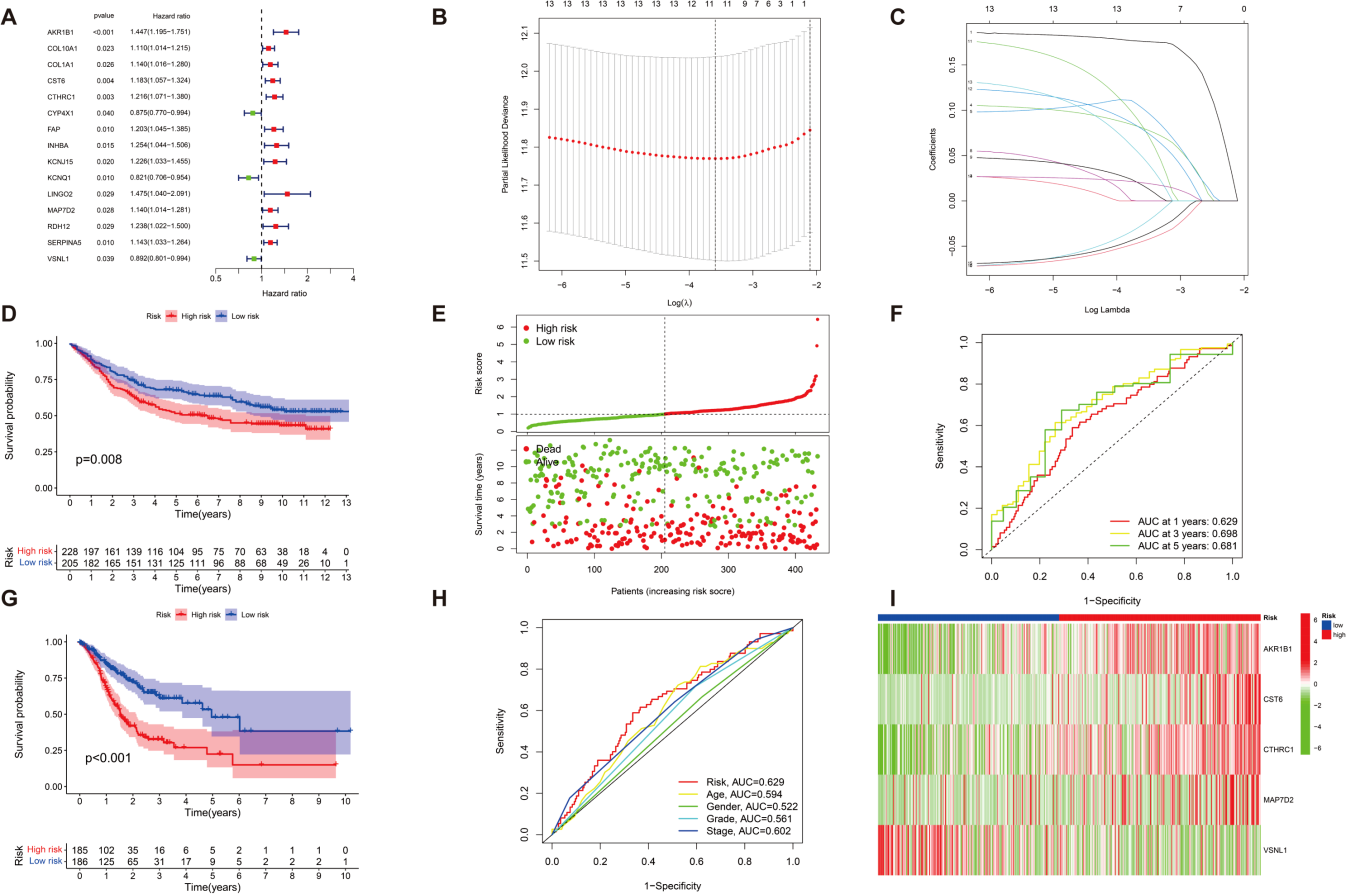
The characterization of prognostic models of DEGs in GC. **(A)** A total of 15 GC-associated genes associated with GC prognosis were drawn on forest plots (*p* < 0.05). **(B, C)** A total of 15 associated GC DEGs verified by (LASSO) Cox regression analysis. **(D, G)** Kaplan–Meier survival analysis comparing high- and low-risk score groups among all patients with GC uncovered that individuals with high risk scores exhibited significantly reduced overall survival compared to those with low-risk scores (*p* < 0.05). **(E)** The risk status of patients with GC is shown by plot of correlation between risk score and survival rate, and patient survival in the high and low survival groups of all GC samples is shown by the scatter plot (*p* < 0.05). **(I)** Heatmap of risk in all GC samples; AKR1B1, CST6, CTHRC1, and MAP7D2 increased with increasing risk scores, and VSNL1 reduced with low risk scores (*p* < 0.05). **(F)** ROC curve (AUC) area predicts the trait of prognostic models for 1-, 3-, and 5-year overall survival. AUC at 1 year: 0.629, 3 years: 0.698, and 5 years: 0.681. **(H)** Calibration ROC curve in the risk plot was 0.629.

We further assessed the predictive reliability of DEGs in assessing the prognosis of patients with GC. After identifying significant clinical trait-associated modules, we extracted hub genes from the most significant brown module (0.65 correlation coefficient, *p* < 0.001), and we further assessed the prognostic value of hub genes in patients with GC by UniCox regression analysis of the TCGA dataset. A total of 15 GC-associated DEGs were found to be associated with GC prognosis by UniCox analysis (*p* < 0.05). AKR1B1, COL10A1, COL1A1, and other related genes (totaling 12 genes) had significantly high expression and were negatively associated with OS of patients with GC (HR>1.1, *p* < 0.05); CYP4X1, KCNQ1, and VSNL1 had significantly low expression and were positively associated with OS of patients with GC (HR < 0.9, *p* < 0.05) (**Figure 4A**). Then, patients were divided into training and test groups. We further analyzed these 15 genes by LASSO Cox regression 10-fold cross-validation and screened out 11 DEGs correlated with GC prognosis (**Figures 4B, C**). We divided patients into high-risk and low-risk groups based on the risk score of each GC patient and respectively used the risk score and survival correlation map, patient survival scatter plot (**Figure 4E**), the correlation heatmap (**Figure 4I**), and the Kaplan–Meier survival curves (**Figures 4D, G**) to evaluate the reliability of the prognostic models. In the scatter plot of patient survival, survival was significantly negatively correlated with risk score (*p* < 0.05). In the correlation heatmap, we found AKR1B1, CST6, CTHRC1, and MAP7D2 with high risk scores, which were associated with lower survival (*p* < 0.05). VSNL1 had low risk scores, which was associated with higher survival (*p* < 0.05). The Kaplan–Meier survival curves showed that patients with high risk scores had significantly shorter survival time than patients with low risk scores. Then, we evaluated the accuracy of prognostic models; the AUC values of patients at 1-year, 3-year, and 5-year survival were all greater than 0.6 in the ROC curves, which makes our prediction more accurate (**Figure 4F**). Finally, we found that the actual 1-year, 3-year, and 5-year survival rates were generally consistent with those predicted by comparing the calibration curves, and the maximum area under the ROC curve in the risk plot was 0.629, which demonstrates the precision of our model in predicting survival in patients with GC (**Figure 4H**).

### Assessing the predictive accuracy of prognostic models in patients with GC

**Figure 5:**
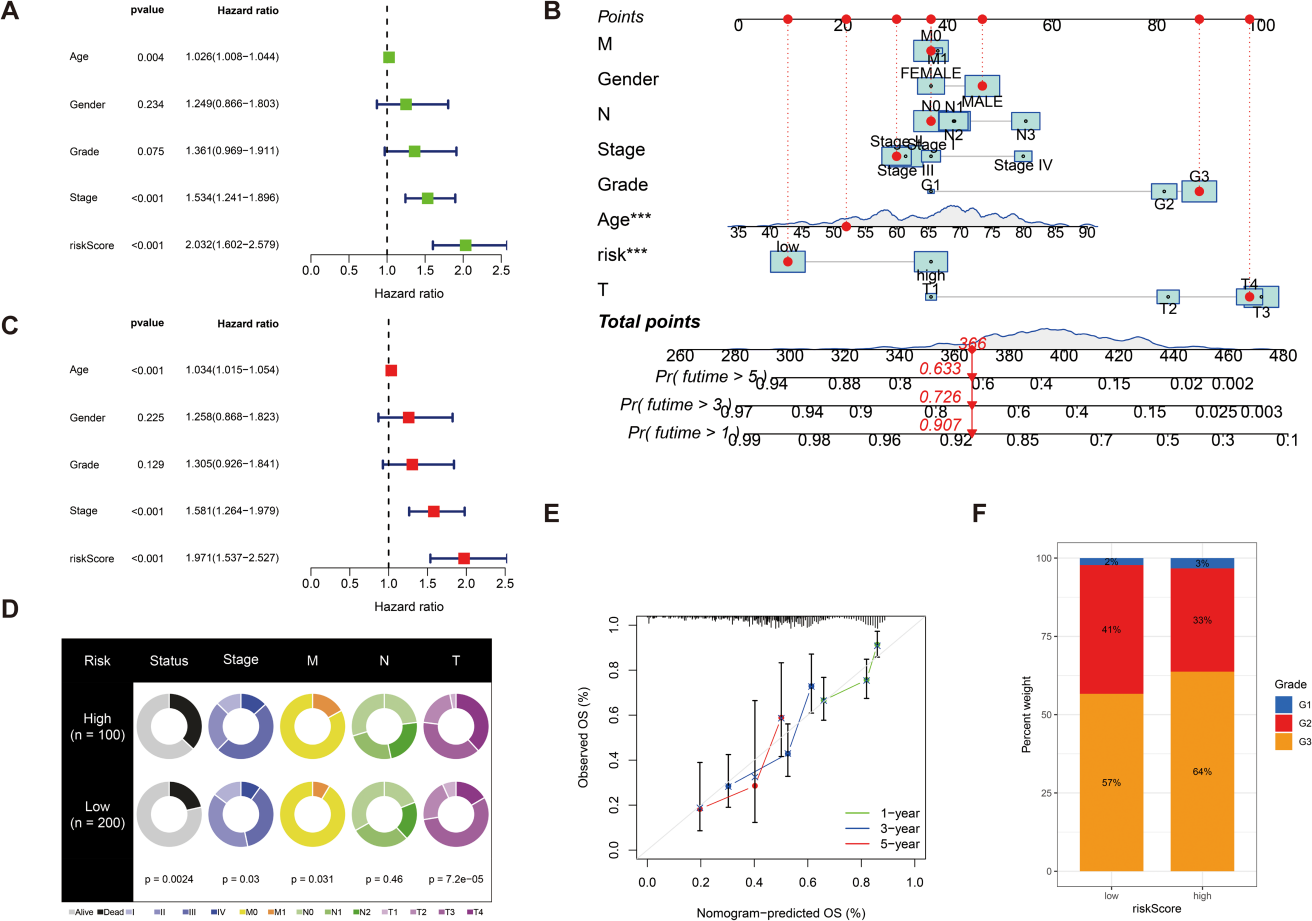
**(A)** Univariate Cox regression analysis of the risk score. **(C)** Multivariate Cox regression analysis of the risk score. **(B)** Line graphs to predict the survival of patients with GC. **(E)** The calibration curve for the nomogram-predicted OS. The *X*-axis is the nomogram-predicted survival and the *Y*-axis is the actual survival, and the calibration curves are almost close to the predicted ones. **(D)** Fan charts to illustrate the variation in risk score expression across different clinical prognostic stages. **(F)** The percent weight of high risk scores and low risk scores in different clinical stages in patients with GC. ***p < 0.001.

We further validated the predictive accuracy of prognostic models, in both UniCox and MultiCox regression analyses, with marked differences; the *p*-values of risk scores were less than 0.001, indicating that risk scores can be an independent prognostic factor for patients with GC, irrespective of other clinical characteristics (**Figures 5A, C**). Next, we constructed line graphs to predict the survival of patients with GC. With a total of 366 patients with GC, our model predicted a 1-year survival rate exceeding 0.907, a 3-year survival rate exceeding 0.726, and a 5-year survival rate exceeding 0.633 for patients with GC; the calibration curves are almost close to the predicted ones (**Figures 5B, E**). Subsequently, we used fan charts to illustrate the variation in risk score expression across different clinical prognostic stages (**Figure 5D**). In different clinical stages in patients with GC, we found that the G1 stage accounts for 2%, the G2 stage accounts for 41%, and the G3 stage accounts for 57% with low risk scores. In high risk scores, the G1 stage accounts for 3%, the G2 stage accounts for 33%, and the G3 stage accounts for 64%; obviously, a greater risk score is associated with a poorer prognosis in patients with GC (**Figure 5F**).

### Immune cell infiltration and microenvironment analysis

**Figure 6:**
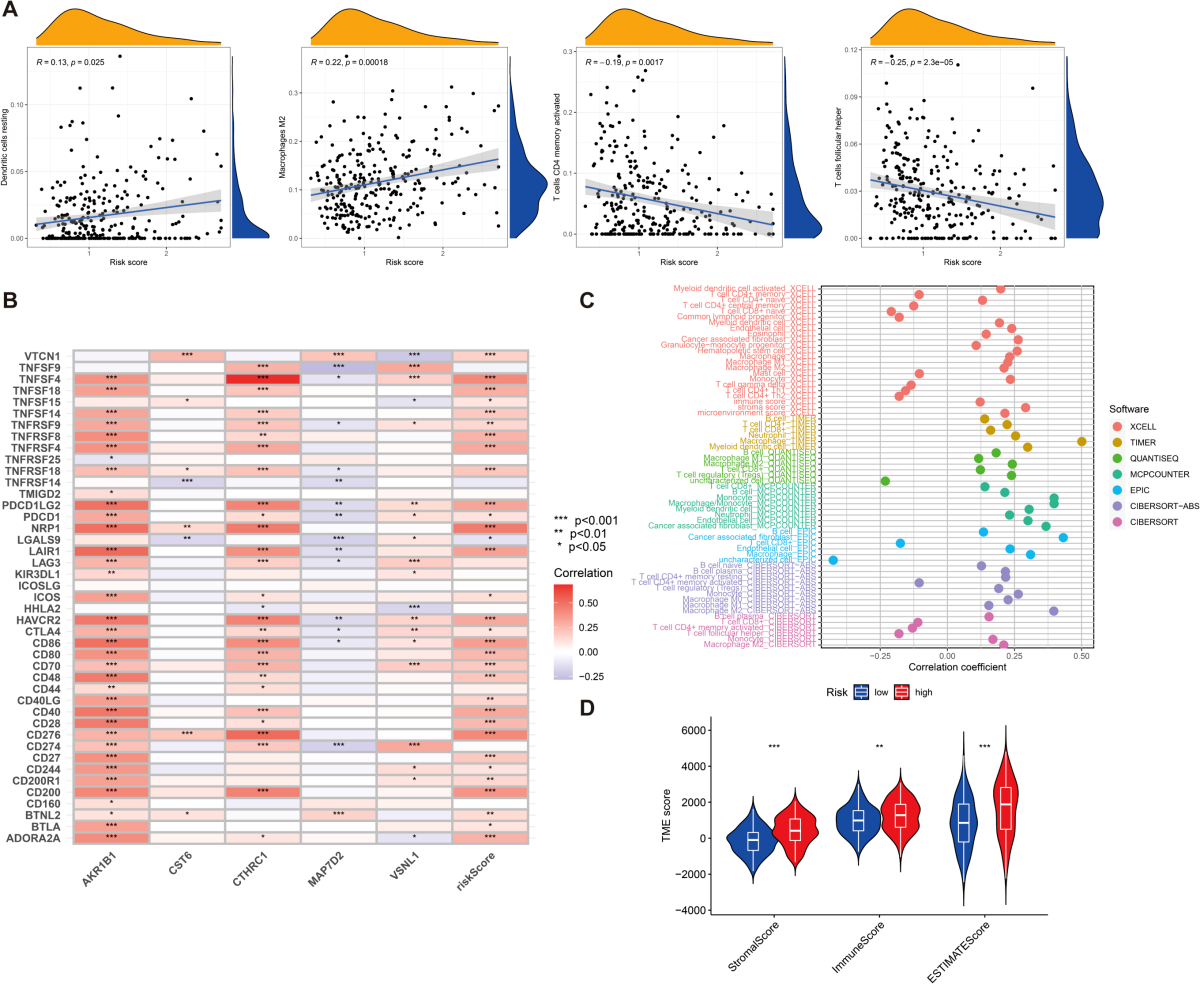
**(A)** The relationship between risk score and various immune cells. **(B)** Correlation between five DEGs, risk score, and 43 immune cells. **(C)** Immune cell bubble plots. **(D)** Differences in stromal, immune, and ESTIMATE scores between high- and low-risk groups were examined. *p < 0.05, **p < 0.01, and ***p < 0.001.

Tumor immune microenvironment plays a crucial role in the development and progression of GC and may influence patient prognosis and response to immunotherapy. Therefore, we sought to explore whether our risk score was also linked to immune cell infiltration, immune checkpoint expression, and immunotherapy response. First, we investigated the tumor immune microenvironment in GC. We found that the expression of resting dendritic cells (DCs) (*R* = 0.13, *p* = 0.025) and M2 macrophages (*R* = 0.22, *p* = 0.00018) was positively correlated with the risk score, and the expression of CD4 memory-activated T cells (*R* = −0.19, *p* = 0.0017) and follicular helper T cells (*R* = −0.25, *p* = 2.3e−05) was negatively correlated with the risk score (**Figure 6A**). We then evaluated the correlations of the five DEGs and risk score used for clustering with 43 immune cells and found that all five genes and risk score of the included genes were significantly associated with at least one immune cell (*p* < 0.05) (**Figure 6B**). The immune cell bubble plots illustrate a stronger correlation between various immune cells on different algorithms (XCELL, TIMER, QUANTISEQ, MCPCOUNTER, EPIC, CIBERSORT-ABS, and CIBERSORT) (**Figure 6C**). Subsequently, we compared the stromal, immune, and ESTIMATE scores between groups with high- and low-risk groups. The results revealed that risk scores had a significant upregulation in high-risk groups (**Figure 6D**) (*p* < 0.001).

**Figure 7:**
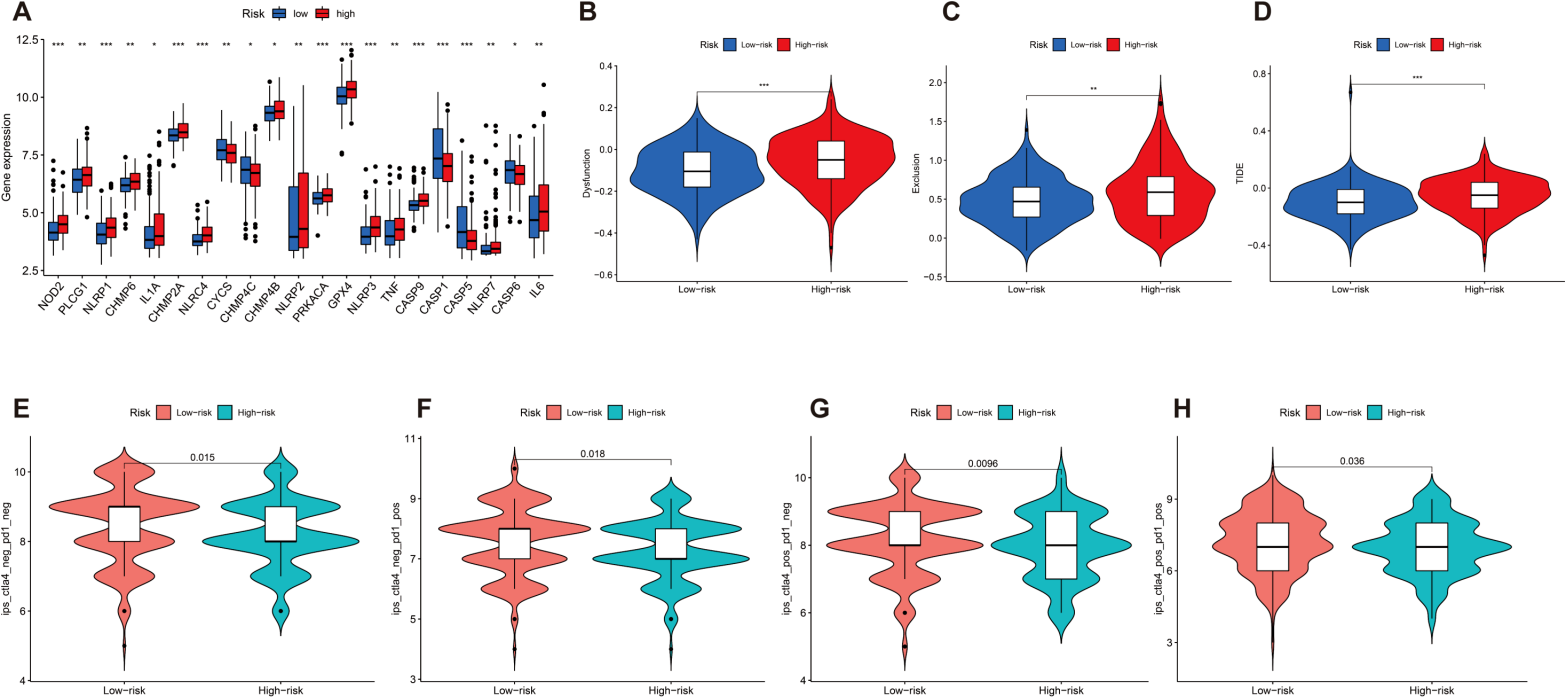
**(A)** Differential expression of 21 immune checkpoint expressions in high- and low-risk groups (*p* < 0.05). **(B–D)** The difference of dysregulation, exclusion, and TIDE scores between high- and low-risk groups (*p* < 0.05). **(E–H)** Violin plot shows the correlation between risk score and immunotherapy (*p* < 0.05). *p < 0.05, **p < 0.01, and ***p < 0.001.

We then investigated the correlation between the expression of 21 immune checkpoint genes and risk scores; some genes are highly expressed in the high-risk score group, such as NOD2, PLCG1, NLRP1, CHMP6, and IL1A, and some genes are highly expressed in the low-risk score group, such as CYCS, CHMP4C, CASP1, CASP5, and CASP6 (*p* < 0.05) (**Figure 7A**). Then, we mapped the different risks on immune cell sensitivity using a violin plot; apparently, we found that dysregulation, exclusion, and TIDE scores were higher in the high-risk groups of patients with GC than in the low-risk groups (*p* < 0.05) (**Figures 7B–D**). Meanwhile, immunotherapy reactivity analysis showed that low expression of risk scores was related to better immunotherapy efficacy of patients with GC; specifically, lower risk scores were associated with improved therapeutic efficacy of PD-1-targeted treatments (*p* < 0.05). This suggests that the risk score may serve as a predictive biomarker for patient stratification in PD-1-based immunotherapy (**Figures 7E–H**).

### Validation of the expression levels of AKR1B1, CST6, and CTHRC1

**Figure 8:**
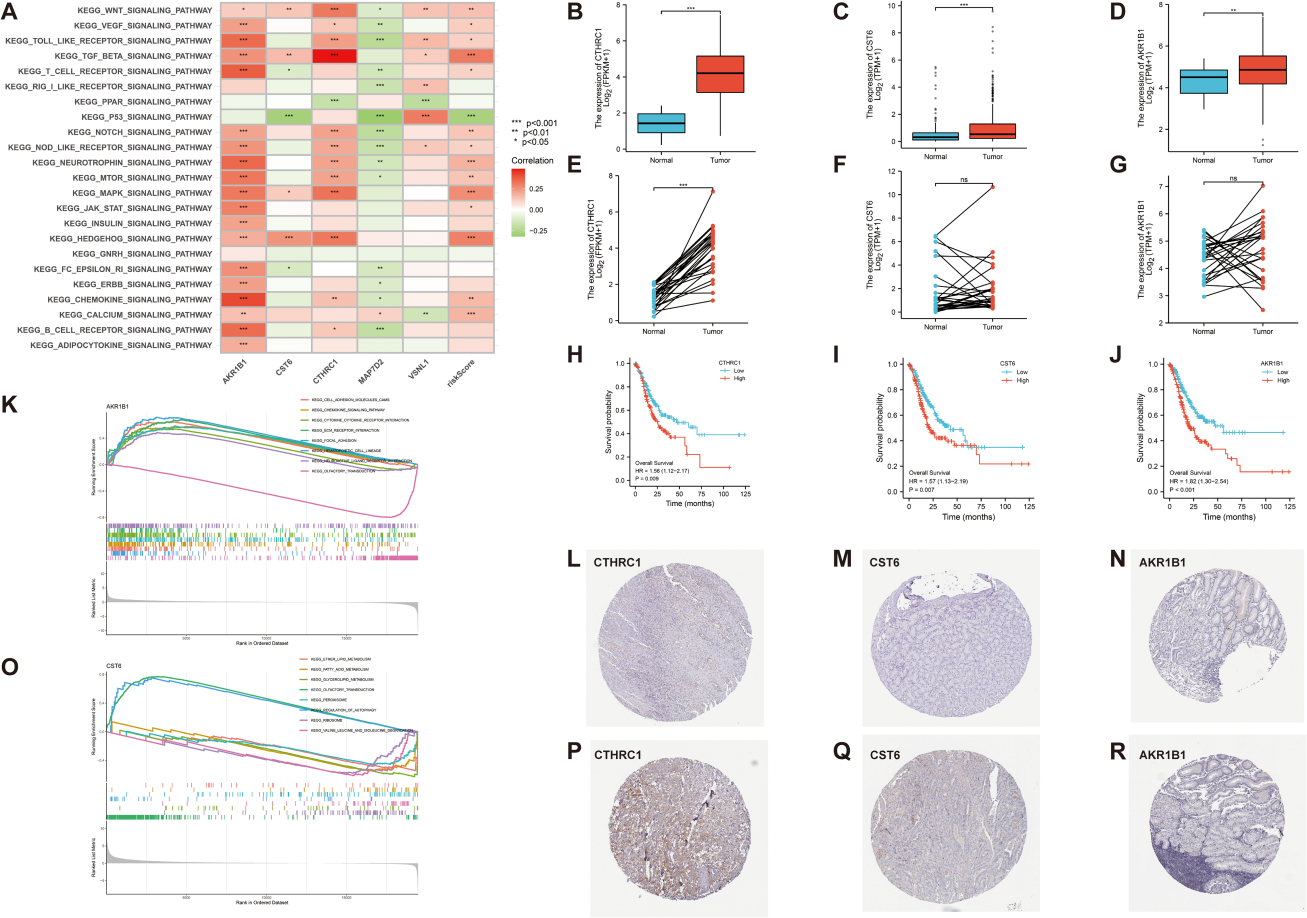
**(A)** Correlation between 23 KEGG pathways and 5 GC-related genes and risk score (*p* < 0.05). **(K, O)** The relationship between KEGG signaling pathways and the high- and low-expression groups of AKR1B1 and CST6 gene. **(B–G)** In both paired and unpaired samples of patients with GC, protein expression levels of CTHRC1, CST6, and AKRIB1 were highly expressed in cancer and lowly expressed in normal samples, with statistically significant differences (*p* < 0.05). **(H–J)** Effect of CTHRC1, CST6, and AKRIB1 expression on the survival of Kaplan–Meier mapper gastric cancer (HR>1.5, *p* < 0.01). **(L–R)** Representative immunohistochemical staining results of CTHRC1, CST6, and AKR1B1 protein in gastric cancer tissue and normal tissue. *p < 0.05, **p < 0.01, and ***p < 0.001.

To further evaluate the robustness and clinical relevance of the 11 DEGs correlated with GC prognosis, we prioritized the top three genes (AKR1B1, CST6, and CTHRC1) with the lowest *p*-values in UniCox regression analysis for a series of validation analyses, and explored their potential therapeutic implications. First, we analyzed the signaling pathways associated with these genes. In our correlation analysis, AKR1B1 exhibits a positive correlation with 19 signaling pathways, including the Wnt, VEGF, and Toll-like receptor signaling pathways (*p* < 0.05). CST6 demonstrates a significant positive correlation with four distinct signaling pathways, including Wnt, TGF-β, MAPK, and HEDGEHOG signaling pathways, and displays a negative correlation with T-cell receptor, P53, and FC-EPSILON-RI signaling pathways (*p* < 0.05). CTHRC1 exhibits a positive correlation with 12 signaling pathways, including Wnt, VEGF, and Toll-like receptor, and displays a negative correlation with PPAR signaling pathways (*p* < 0.05). Next, we found that risk score demonstrates a significant positive correlation with 14 signaling pathways, such as Wnt, TGF-β, and MAPK signaling pathways, and displays a negative correlation with P53 signaling pathways (*p* < 0.05) (**Figure 8A**). In the KEGG enrichment score, a total of seven signaling pathways, including cell adhesion molecules cams, chemokine-signaling pathway, and other related pathways in cancer, are active in the high AKR1B1 expression group, while olfactory transduction is active in the low AKR1B1 expression group. Analogously, the KEGG pathways olfactory transduction and regulation of autophagy are active in the high CST6 expression group, while ether lipid metabolism, fatty acid metabolism, glycerolipid metabolism, peroxisome, ribosome, and valine, leucine, and isoleucine degradation are active in the low CST6 expression group (**Figures 8K, O**). Among the GC-related genes, CTHRC1, CST6, and AKR1B1 were significantly differentially expressed in GC samples from the GEO database. With statistically significant differences, CTHRC1, CST6, and AKR1B1 were highly expressed in the tumor and lowly expressed in the normal in both paired and unpaired samples of patients with GC (*p* < 0.05) (**Figures 8B–G**). Elevated gene expression demonstrated a significant correlation with prognosis, with higher expression levels being associated with markedly poorer clinical outcomes (HR>1.5, *p* < 0.01) (**Figures 8H–J**). To validate the protein-level expression of the prognostic biomarkers, we used IHC staining data from the HPA database for AKR1B1, CST6, and CTHRC1, which were identified as key genes in our prognostic model. The results showed that the protein levels were significantly elevated in GC tissues compared to normal tissues (**Figures 8L–R**), indicating that these three GC-associated genes have the potential to serve as tumor biomarkers.

### GSEA results for CTHRC1, CST6, and AKR1B1

**Figure 9:**
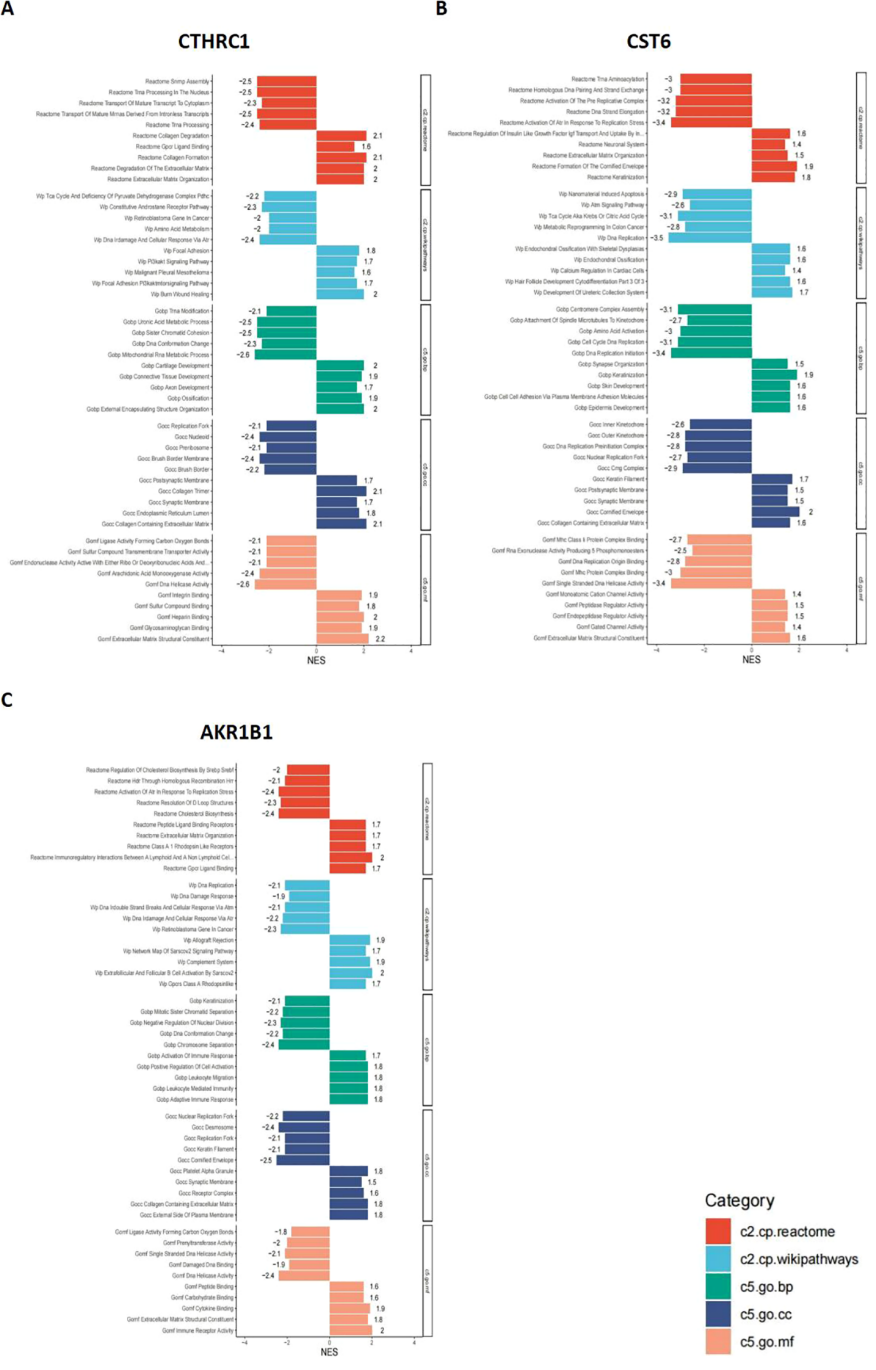
GSEA results for CTHRC1, CST6, and AKR1B1. GSEA was performed to identify signaling pathways associated with the expression of **(A)** CTHRC1, **(B)** CST6, and **(C)** AKR1B1. The bar plots show the top significantly enriched pathways (|NES| > 1, *p* < 0.05), grouped by functional category. The *X*-axis indicates the NES. Pathway categories are color-coded: red indicates the c2.cp.reactome gene sets, light blue represents c2.cp.wikipathways, green corresponds to c5.go.bp, dark blue denotes c5.go.cc, and orange refers to c5.go.mf.

In this study, bar plots with distinct colors were used to represent different categories of gene sets. Specifically, red indicates the c2.cp.reactome gene sets, light blue represents c2.cp.wikipathways, green corresponds to c5.go.bp, dark blue denotes c5.go.cc, and orange refers to c5.go.mf. The direction of each bar reflects the enrichment pattern of the gene set in the sample groups: bars extending to the left indicate significant enrichment in the low-expression group, while bars extending to the right indicate significant enrichment in the high-expression group. As shown in **Figures 9A–C**, the CTHRC1 GSEA revealed significant enrichment of multiple biological pathways. Specifically, pathways associated with collagen degradation (NES = 2.1, *p* < 0.05), degradation of the extracellular matrix (NES = 2, *p* < 0.05), and extracellular matrix structural constituent (NES = 2.2, *p* < 0.05) were significantly upregulated, and pathways associated with tRNA processing in the nucleus (NES = −2.5, *p* < 0.05), DNA damage and cellular response via Atr (NES = −2.4, *p* < 0.05), and mitochondrial RNA metabolic process (NES = −2.6, *p* < 0.05) were significantly downregulated. The CST6 GSEA revealed that pathways associated with cornified envelope (NES = 2, *p* < 0.05), cell–cell adhesion via plasma membrane adhesion molecules (NES = 1.6, *p* < 0.05), and extracellular matrix structural constituent (NES = 1.6, *p* < 0.05) were significantly upregulated, and pathways associated with activation of Atr in response to replication stress (NES = −3.4, *p* < 0.05), DNA replication (NES = −3.5, *p* < 0.05), and DNA replication initiation (NES = −3.4, *p* < 0.05) were significantly downregulated. The AKR1B1 GSEA revealed that pathways associated with immunoregulatory interactions between lymphoid and non-lymphoid cells (NES = 2, *p* < 0.05), extracellular matrix organization (NES = 1.7, *p* < 0.05), and cytokine binding (NES = 1.9, *p* < 0.05) were significantly upregulated, and pathways associated with activation of Atr in response to replication stress (NES = −2.4, *p* < 0.05), cholesterol biosynthesis (NES = −2.4, *p* < 0.05), and DNA replication (NES = −2.1, *p* < 0.05) were significantly downregulated.

These results indicate that CTHRC1, CST6, and AKR1B1 high expression are characterized by enhanced immune activity and upregulation of extracellular matrix organization, accompanied by suppression of DNA repair and cell division-related pathways. Such a shift may reflect a remodeling of the tumor immune microenvironment or a disruption of cellular homeostasis, potentially contributing to tumor progression or therapeutic response heterogeneity.

### Evaluation of the prognostic and survival analysis for CTHRC1, CST6, and AKR1B1

**Figure 10:**
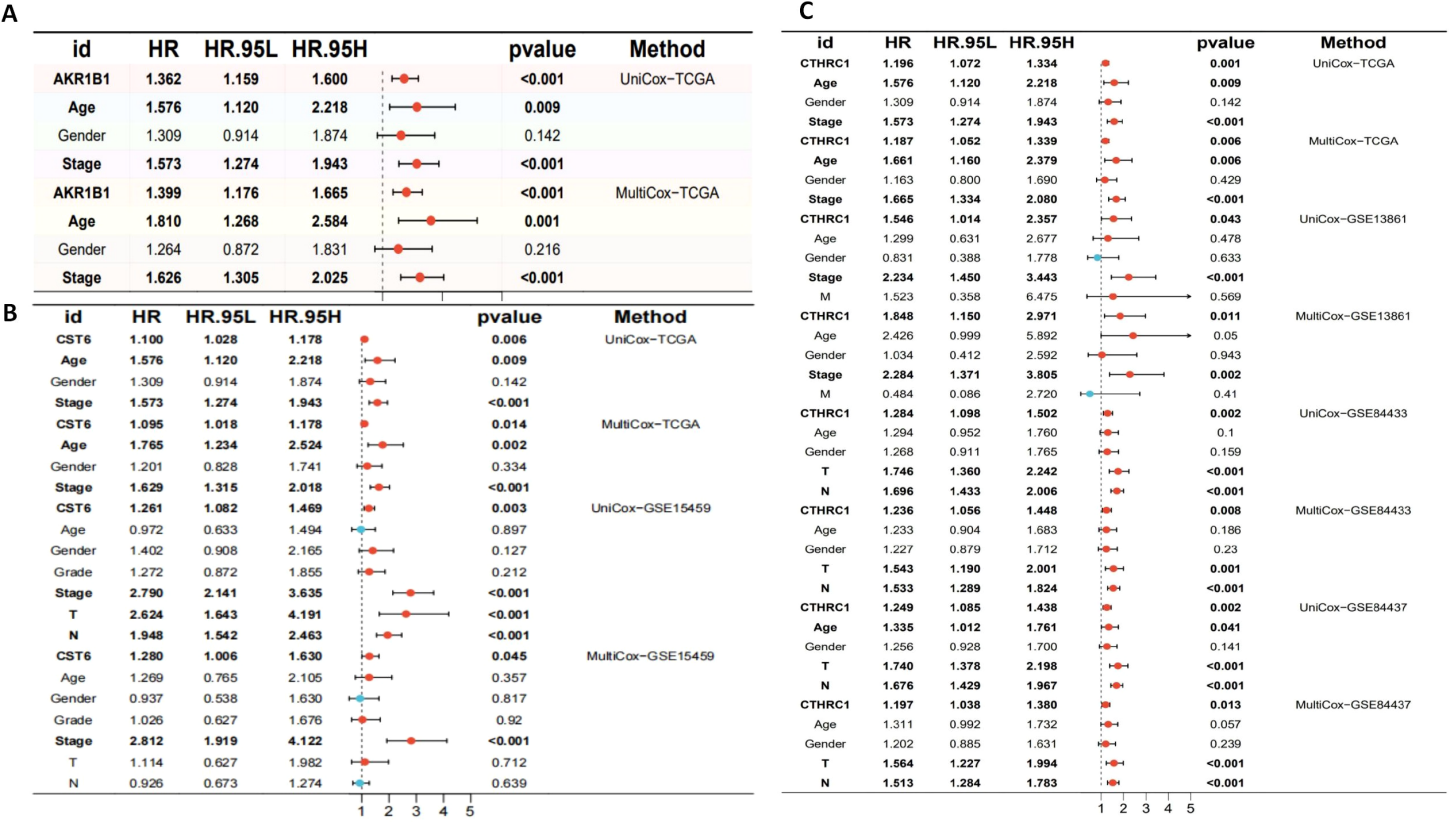
Univariate and multivariate Cox regression analyses of AKR1B1, CST6, and CTHRC1 in gastric cancer across multiple cohorts. **(A)** Univariate and multivariate Cox regression analyses of AKR1B1 in the TCGA cohort. **(B)** Cox regression results for CST6 in the TCGA and GSE15459 datasets. **(C)** Cox regression analyses for CTHRC1 in the TCGA, GSE13861, GSE84433, and GSE84437 cohorts. HR, 95% CI, and *p*-values are presented. Red dots indicate variables with HR > 1, while blue dots represent HR < 1. Statistically significant results (*p* < 0.05) are emphasized. Both UniCox and MultiCox methods were used to assess the prognostic value of gene expression and clinical parameters.

The aberrant expression of these three genes correlates with clinical outcomes in GC. Therefore, comprehensive survival and prognostic analyses of these genes are essential to elucidate their potential clinical utility. We visualized the results of UniCox survival analysis performed using the “survival” package on gene expression (CTHRC1, CST6, and AKR1B1) and conventional clinical variables (age, gender, and stage). CTHRC1, CST6, and AKR1B1 are all associated with poor survival prognosis (HR > 1, *p* < 0.05), with CTHRC1 consistently identified across multiple datasets. Similarly, MultiCox regression analysis revealed that the expression levels of CTHRC1, CST6, and AKR1B1, along with certain clinical variables, were significantly associated with poor survival outcomes (HR > 1, *p* < 0.05). Notably, even after adjusting for other clinical factors, these genes remained statistically significant (*p* < 0.05), indicating that AKR1B1, CST6, and CTHRC1 are independent prognostic factors potentially critical to patient survival (**Figures 10A–C**).

### Single-cell expression levels of CTHRC1, CST6, and AKR1B1in gastric cancer tissues

**Figure 11:**
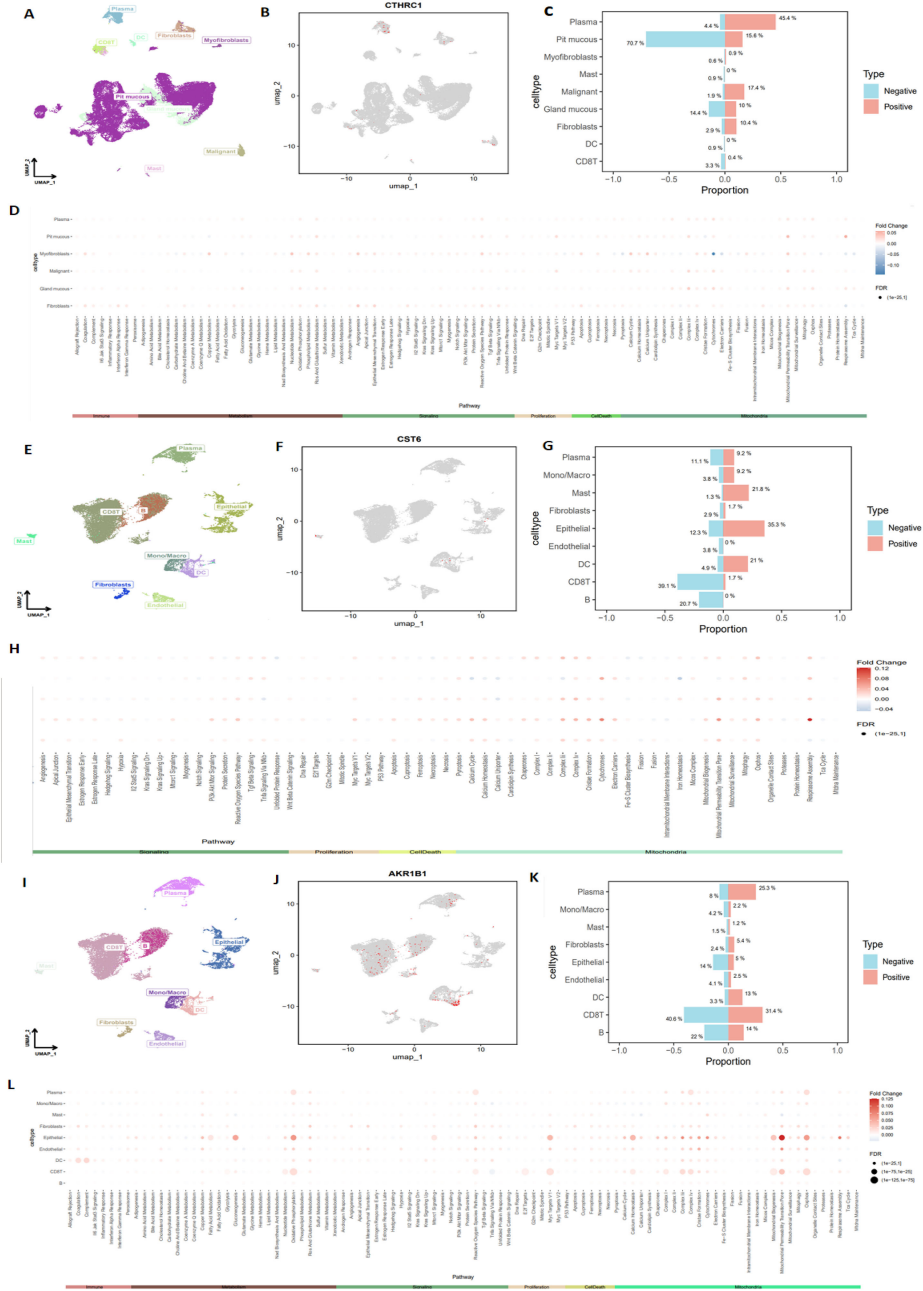
Detailed analysis of CTHRC1, CST6, and AKR1B1 expression in STAD, using single-cell sequencing data. **(A, B)** Single-cell, single-gene UMAP visualization highlighting significant variability in CTHRC1 expression across different cell clusters within STAD, **(C)** with notable expression in plasma, pit mucous, and malignant cells. **(D)** AUCell scoring of various biological pathways in STAD, showing higher mitochondria-related pathway scores in cells positively expressing CTHRC1 (−0.1 < logFC < 0.05, FDR < 0.01). **(E, F)** Single-cell, single-gene UMAP visualization highlighting significant variability in CST6 expression across different cell clusters within STAD, **(G)** with notable expression in epithelial, mast, and DC. **(H)** AUCell scoring of various biological pathways in STAD, showing higher mitochondria-related pathway scores in cells positively expressing CST6 (−0.04 < logFC < 0.12, FDR < 0.01). **(I, J)** Single-cell, single-gene UMAP visualization highlighting significant variability in AKR1B1 expression across different cell clusters within STAD, **(K)** with notable expression in CD8 T cells, plasma, and B cells. **(L)** AUCell scoring of various biological pathways in STAD, showing higher mitochondria-related pathway scores in cells positively expressing AKR1B1 (0 < logFC < 0.125, FDR < 0.01).

To further investigate the types of cells expressing CTHRC1, CST6, and AKR1B1 in tumor tissues, we utilized the GEO database datasets EMTAB8107 and GSE167297 to conduct a detailed analysis of CTHRC1, CST6, and AKR1B1 expression in STAD. Through single-cell, single-gene UMAP visualization (**Figures 11A, B**), we observed significant variability in CTHRC1 expression across different tissue components within STAD, showing positive expression in plasma, pit mucous, and malignant cells, and negative expression in pit mucous, gland mucous, and plasma cells (**Figure 11C**). Additionally, using the AUCell package to score various biological pathways, we found higher mitochondria-related pathway scores in cells positively expressing CTHRC1 (−0.1 < logFC < 0.05, FDR < 0.01) (**Figure 11D**). Similarly, through CST6-related single-cell, single-gene UMAP visualization (**Figures 11E, F**), we found significant variability in CST6 expression across different tissue components within STAD, particularly positively expressed in epithelial cells, mast cells, and DCs, and negatively expressed in CD8 T cells, B cells, and plasma (**Figure 11G**). We also observed higher mitochondria-related pathway scores in cells positively expressing CST6 (−0.04 < logFC < 0.12, FDR < 0.01) (**Figure 11H**). In AKR1B1, we also applied single-cell, single-gene UMAP visualization (**Figures 11I, J**), showing positive expression in CD8 T cells, plasma, and B cells, and negative expression in CD8 T cells, B cells, and epithelial cells (**Figure 11K**), and in cells positively expressing AKR1B1, scores related to mitochondria pathways were also higher (0 < logFC < 0.125, FDR < 0.01) (**Figure 11L**). These results indicate that CTHRC1, CST6, and AKR1B1 expression exhibits significant variability in GC, especially within key immune cell populations such as CD8 T cells, B cells, and DCs. Moreover, our data suggest a potential crucial role for CTHRC1, CST6, and AKR1B1 in the mitochondria process of tumor cells. These findings offer critical insights to further investigate the potential roles of CTHRC1, CST6, and AKR1B1 within the tumor immune microenvironment.

### Validation of CTHRC1, CST6, and AKR1B1 expression levels in clinical gastric cancer tissue samples by quantitative real-time polymerase chain reaction

**Figure 12:**
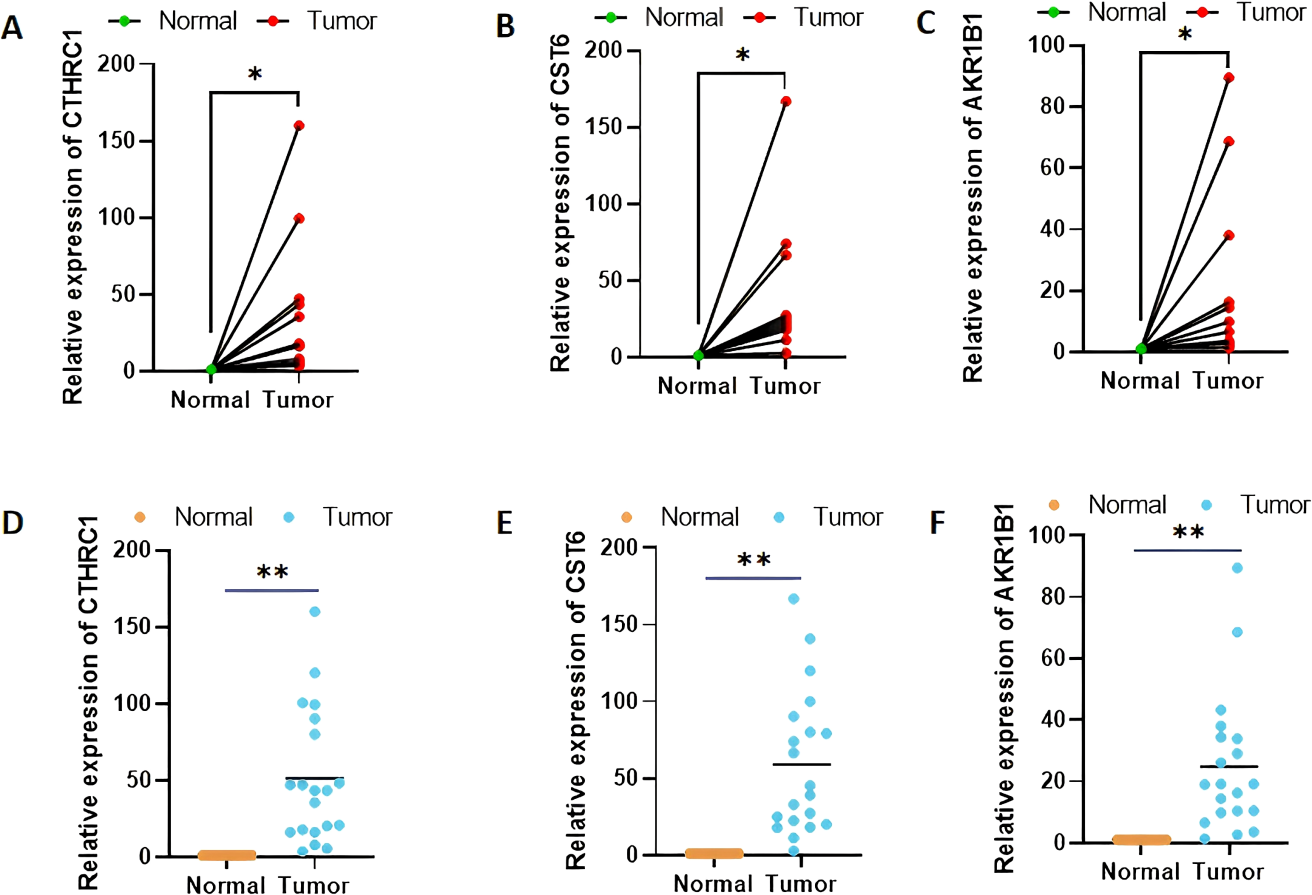
Validation of CTHRC1, CST6, and AKR1B1 expression levels in clinical tissue samples by quantitative real-time polymerase chain reaction **(A–F)**. *p < 0.05, **p < 0.01.

Among the three genes with prognostic characteristics, the expression levels in GC and adjacent normal tissues were detected by qRT-PCR; CTHRC1, CST6, and AKR1B1 were all more highly expressed in GC tumor tissues (*p* < 0.05) (**Figures 12A–F**).

## Discussion

GC represents one of the most common malignancies worldwide, characterized by significant molecular and phenotypic heterogeneity with persistently high mortality rates despite therapeutic advancements (3, 4). The complex pathogenesis involves genetic mutations, chromosomal abnormalities, differential gene expression, and epigenetic modifications that create a tumor microenvironment facilitating immune evasion and treatment resistance (13–15). Therefore, understanding these molecular characteristics and immunosuppressive mechanisms is essential for developing innovative therapeutic strategies (5–7). Our research demonstrates that molecular clustering and prognostic features derived from integrated TCGA and GEO databases can effectively predict patient outcomes and characterize the immune landscape in GC. We identified two distinct PRG clusters and established an 11-gene signature that stratified patients into high-risk and low-risk groups with significant differences in survival outcomes and immune profiles. Through comprehensive bioinformatics analysis, we identified 11 DEGs significantly associated with GC prognosis, with CTHRC1, CST6, and AKR1B1 selected for experimental validation based on their strong prognostic significance. The risk score calculated from these DEGs demonstrated robust predictive power for patient survival outcomes, consistent with approaches used by other researchers developing molecular signatures for cancer prognosis (16, 17).

Based on the identified prognostic genes, we constructed a risk score model for predicting survival outcomes in patients with GC. The Kaplan–Meier survival analysis revealed that patients with high risk scores had significantly poorer OS compared to those with low risk scores across both training and validation cohorts, with our model achieving solid predictive accuracy (AUC values of 0.629, 0.698, and 0.681 for 1-year, 3-year, and 5-year survival, respectively). The model’s predictive performance was rigorously evaluated using several approaches. The UniCox and MultiCox regression analyses confirmed that the risk score is an independent prognostic factor (*p* < 0.001), independent of age, gender, grade, and TNM stage, even after adjusting for established clinicopathological features. To enhance clinical applicability, a nomogram incorporating the risk score was developed to predict individual patient survival probabilities, exhibiting excellent predictive performance as evidenced by calibration curves that showed good agreement between predicted and actual survival rates. In a cohort of 366 patients with GC, the model predicted 1-year, 3-year, and 5-year survival probabilities of >0.907, >0.726, and >0.633, respectively, outperforming other clinical features in prognostic capability.

Our analysis also revealed significant associations between risk scores and immune cell infiltration patterns within the tumor microenvironment. Specifically, we observed positive correlations between risk scores and both resting DCs (*R* = 0.13, *p* = 0.025) and M2 macrophages (*R* = 0.22, *p* = 0.00018), while activated CD4 memory T cells (*R* = −0.19, *p* = 0.0017) and follicular helper T cells (*R* = −0.25, *p* = 2.3e−05) showed negative correlations. Additionally, we found that risk scores correlated with the expression of important immune checkpoint genes (including PD-1, CTLA-4, and LAG3), and TIDE analysis showed higher scores for dysregulation, exclusion, and overall TIDE in the high-risk group, suggesting potential utility in predicting immunotherapy responses. To validate our computational findings, we performed experimental validation using qRT-PCR, confirming significant overexpression of CTHRC1, CST6, and AKR1B1 in GC tissues compared to adjacent normal tissues (*p* < 0.05), with HPA data further confirming elevated protein levels in tumor tissues. We further investigate the types of cells expressing CTHRC1, CST6, and AKR1B1 in tumor tissues, and the results indicate that CTHRC1, CST6, and AKR1B1 expression exhibits significant variability in GC, especially within key immune cell populations such as CD8 T cells, B cells, and DCs. Existing literature demonstrates that AKR1B1 has been reported to modulate reactive oxygen species and inflammatory responses, which could affect immune cell recruitment and function in the tumor microenvironment (18). CST6, as a cysteine protease inhibitor, may influence antigen presentation and extracellular matrix remodeling. By inhibiting cathepsin activity, CST6 could alter DC maturation and function, thereby affecting T cell priming and infiltration (19). In addition, CTHRC1 may indeed play a functional role in shaping the tumor immune microenvironment. Recent studies suggest that CTHRC1 has been shown to enhance the recruitment and polarization of tumor-associated macrophages toward an M2 phenotype, which is commonly associated with immunosuppression and tumor progression. For example, Zhuo et al. (20) demonstrated that CTHRC1 can activate the PI3K-Akt signaling pathway and modulate cytokine secretion (IL-10 and TGF-β), facilitating M2 macrophage polarization and immune escape. Taken together, our prognostic model offers several valuable clinical applications, including the identification of high-risk patients requiring more aggressive treatment or closer follow-up, the selection of patients likely to respond to immunotherapy based on immune infiltration patterns, and the identification of potential therapeutic targets based on dysregulated genes and pathways, with our nomogram incorporating both molecular and clinical characteristics demonstrating good calibration between predicted and actual survival probabilities.

Our findings were validated against relevant published literature, demonstrating consistent results with prior studies. CTHRC1 is a secreted glycoprotein that functions as an extracellular matrix protein involved in tissue remodeling, wound healing, and cell migration processes (21). It has been identified as a critical regulator of multiple signaling pathways relevant to cancer. CTHRC1 is notably upregulated in gastric carcinoma tissues compared to normal gastric tissues, with its elevated expression correlating with advanced stages of the disease, including deeper tumor invasion and lymph node metastasis. This overexpression is associated with poorer overall and disease-free survival in patients with GC, highlighting CTHRC1 as a significant independent prognostic marker (22). Furthermore, CTHRC1 has been shown to enhance GC metastasis via the HIF-1α/CXCR4 signaling pathway; it upregulates CXCR4 expression through HIF-1α and facilitates the migration and invasion of cancer cells, promoting tumor spread to distant organs, thus playing a crucial role in metastasis (23). Some studies indicated that the expression of CTHRC1 is also modulated by epigenetic changes, such as promoter demethylation, which can be reversed by treatment with demethylating agents, suggesting that CTHRC1’s upregulation is linked to both tumor progression and metastasis (24). Additionally, TGF-β1 has been identified as a key regulator that enhances CTHRC1 expression, further contributing to the aggressive nature of GC (25). Given its significant correlation with tumor aggressiveness, high CTHRC1 expression serves as a reliable predictor of poor prognosis in patients with GC, making it a valuable biomarker for early detection and a potential therapeutic target. In conclusion, CTHRC1 plays a pivotal role in the progression and metastasis of GC, through promoting tumor invasion, regulating immune cell infiltration, and enhancing angiogenesis. Its expression levels not only serve as an important prognostic indicator but also present a promising therapeutic target to improve patient outcomes in GC (26).

CST6 is a cysteine protease inhibitor that demonstrates complex and context-dependent functions in cancer pathogenesis (27–29). In our analysis, we observed significant upregulation of CST6 in GC tissues compared to normal tissues. Our qRT-PCR validation confirmed the significant upregulation of CST6 in GC tumor tissues compared to adjacent normal tissues (*p* < 0.05), supporting its potential as a diagnostic marker. As supported by Xu et al. (30), CST6 might play a tumor-promoting role by contributing to immune evasion and facilitating metastatic processes. Our pathway analysis indicated that CST6 expression was significantly associated with olfactory transduction and autophagy regulation, while low expression was related to various metabolic pathways, including lipid and amino acid metabolism. Interestingly, some previous studies have reported contrasting findings regarding CST6 expression in GC. For instance, Lalmanach et al. (28) identified cases where CST6 was downregulated in certain GC subtypes, with epigenetic silencing through DNA methylation in its promoter region being proposed as one mechanism contributing to its reduced expression. Similarly, Qiu et al. (27) also demonstrated that CST6 gene silencing can occur in GC due to promoter hypermethylation. The apparent contradiction between these findings and our results highlights the complex and possibly subtype-dependent role of CST6 in GC biology. The dysregulation of CST6-associated pathways in high-risk patients may explain the varied prognosis associated with altered CST6 expression observed across different studies. The expression heterogeneity of CST6 across different GC studies and tumor types suggests its potential as a novel target for further research.

AKR1B1, an NADPH-dependent oxidoreductase, plays a multifaceted role in GC through several interlinked molecular mechanisms. It modulates oxidative stress by reducing aldehydes and ketones, thereby disrupting cellular redox balance and triggering inflammatory signaling pathways that promote tumorigenesis (31). Moreover, AKR1B1 is implicated in prostaglandin metabolism, particularly in the synthesis of PGF2α, which stimulates cell proliferation, while also modulating pro-apoptotic pathways activated by agents such as 15-deoxy-PGJ2, collectively influencing tumor cell survival and proliferation (32). In addition, its overexpression was found to be associated with increased immune cell infiltration, suggesting a role in remodeling the tumor microenvironment and potentially impacting the efficacy of immunotherapeutic strategies (33). Furthermore, AKR1B1 is involved in regulating gene expression at both transcriptional and post-transcriptional levels, affecting key genes involved in metastasis and cell survival; this dysregulation correlates with adverse clinicopathological features including larger tumor size, lymph node metastasis, and advanced TNM stages, ultimately serving as an indicator of poor prognosis (34, 35). Collectively, these findings underscore the critical involvement of AKR1B1 in GC progression and highlight its potential as a prognostic biomarker and therapeutic target.

Compared with traditional TCGA molecular classifications, which are biologically driven taxonomies derived from unsupervised clustering of multi-omics data, our prognostic models employ machine learning or methods (like Cox regression) to estimate clinical outcomes like survival or treatment response, requiring rigorous validation of predictive performance and generating actionable risk scores for clinical decision-making. In contrast, the TCGA classification primarily serves mechanistic investigations, with prognostic value being a secondary finding. However, in practical implementation, prognostic models and TCGA classification serve distinct but complementary roles in cancer research, TCGA offers raw data for analysis, and prognostic models synthesize these data into actionable insights. The current integrative approaches often benchmark novel prognostic models against established TCGA classifications to enhance both biological interpretability and clinical utility.

Several limitations should be acknowledged, and their implications for clinical translation must be carefully considered: (1) In our study, we utilized retrospective data analyses, offering important insights into the molecular landscape of GC, but they also come with significant limitations. One of the primary concerns is the potential for bias inherent in the use of pre-existing datasets. Retrospective studies often rely on historical clinical data, which may not always represent the full spectrum of the disease or capture the full genetic and phenotypic diversity of the patient population. The datasets we used, such as those from TCGA and GEO, focus on specific cohorts of patients with GC that may not be fully representative of the global GC population, particularly when considering differences in ethnic backgrounds, environmental factors, and disease stages. Additionally, retrospective datasets often suffer from incomplete patient information, missing clinical data, or inadequate follow-up, which limits the ability to assess causality or to explore the temporal dynamics of gene expression and disease progression. (2) Without experimental validation, we cannot conclusively determine the exact mechanisms by which these genes contribute to the altered tumor phenotype. For example, certain genes might regulate immune cell infiltration, potentially influencing tumor growth or the response to therapy. These immune cell interactions could affect key signaling pathways involved in cell proliferation, differentiation, and apoptosis, all of which are pivotal for maintaining tumor homeostasis. However, computational analyses alone cannot clarify how these pathways are activated or inhibited in the context of GC. To understand these processes, functional validation is necessary to experimentally test the role of the identified genes in modulating these cellular processes. (3) We cannot confirm the translational potential of these findings in clinical settings without validation. While the associations with prognosis and immune status are promising, the lack of functional data means that these biomarkers cannot yet be reliably used to guide therapeutic decisions or predict treatment efficacy.

Given these limitations, future experimental studies are essential to validate the functional roles of these genes in GC. We plan to conduct experimental validation *in vitro* and *in vivo* to confirm the impact of these genes on key cellular processes such as cell proliferation, apoptosis, and epithelial–mesenchymal transition, as well as their involvement in immune modulation. Such studies will enable us to better understand how these genes contribute to tumor progression and immune evasion and assess their potential as therapeutic targets. Ultimately, these experimental findings will provide the necessary evidence to translate our bioinformatics predictions into clinically actionable insights.

## Conclusion

Our study established a robust prognostic model based on molecular clustering and gene expression profiles that effectively predicts survival and immune status in patients with GC. The identified genes (CTHRC1, CST6, and AKR1B1) and their associated pathways provide insights into GC progression mechanisms and could serve as high-priority candidates for further validation as predictive biomarkers. Simultaneously, the process of gene screening highlights the value of integrating multi-omics data (e.g., TCGA/GEO databases) to improve biomarker robustness, reducing false positives common in single-gene biomarkers. Our prognostic model’s association with PD-1 signaling suggests their potential utility in stratifying patients likely to respond to ICIs. In addition, these findings could be adapted into targeted next-generation sequencing and other technologies for clinical application, and the strong association between our risk model and immune infiltration patterns suggests its utility in guiding immunotherapy decisions, potentially contributing to more effective personalized treatment strategies for patients with GC.

## Data Availability

The datasets presented in this study can be found in online repositories. The names of the repository/repositories and accession number(s) can be found in the article/**Supplementary Material**.
