# Supplementary material for: Molecular clustering and prognostic features based on integrated databases predict survival and immune status in patients with gastric cancer
Source: Front Oncol. 2025 Sep 4;15:1642911. doi: 10.3389/fonc.2025.1642911 (PMC12444767; doi:10.3389/fonc.2025.1642911)
Supplement: Supplementary file 1 [file DataSheet1.docx]

**Figure S1**

**
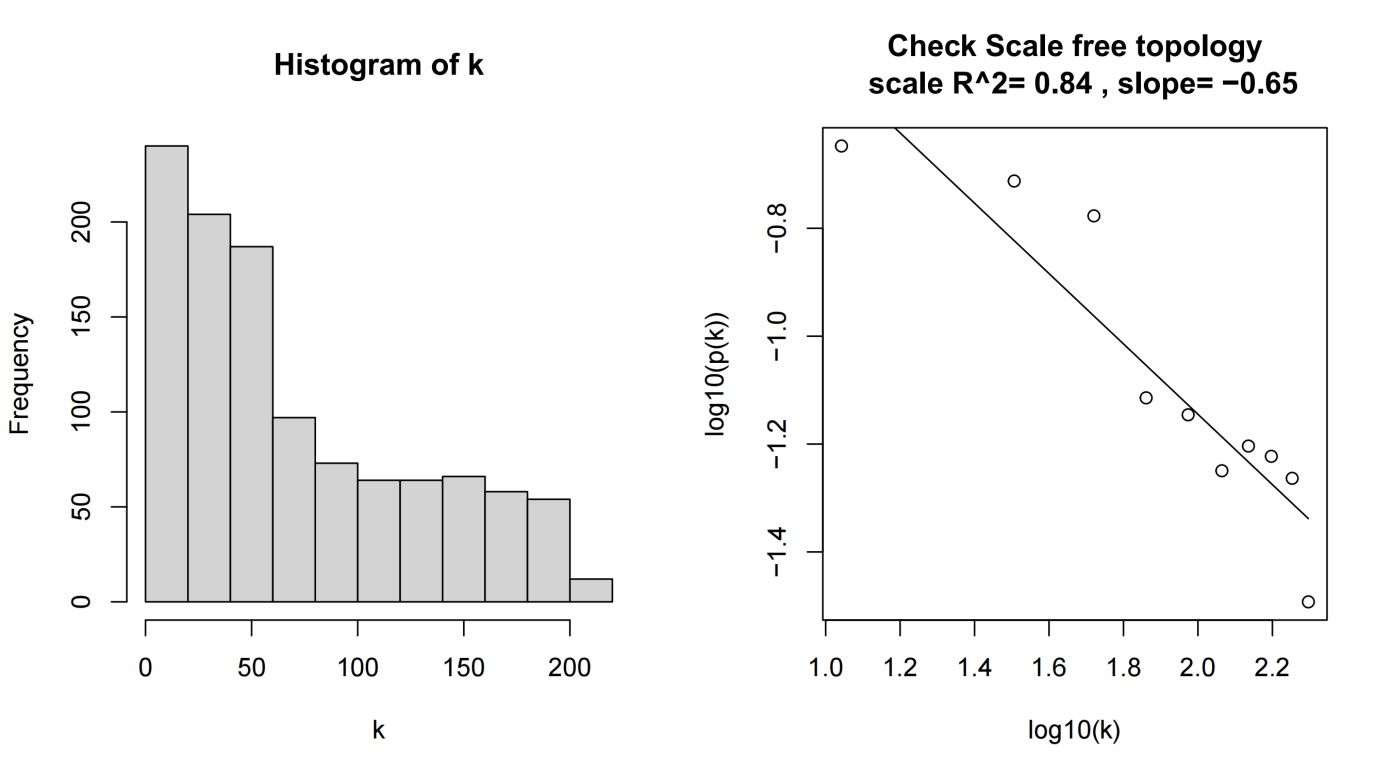
**

**Figure S2**

**
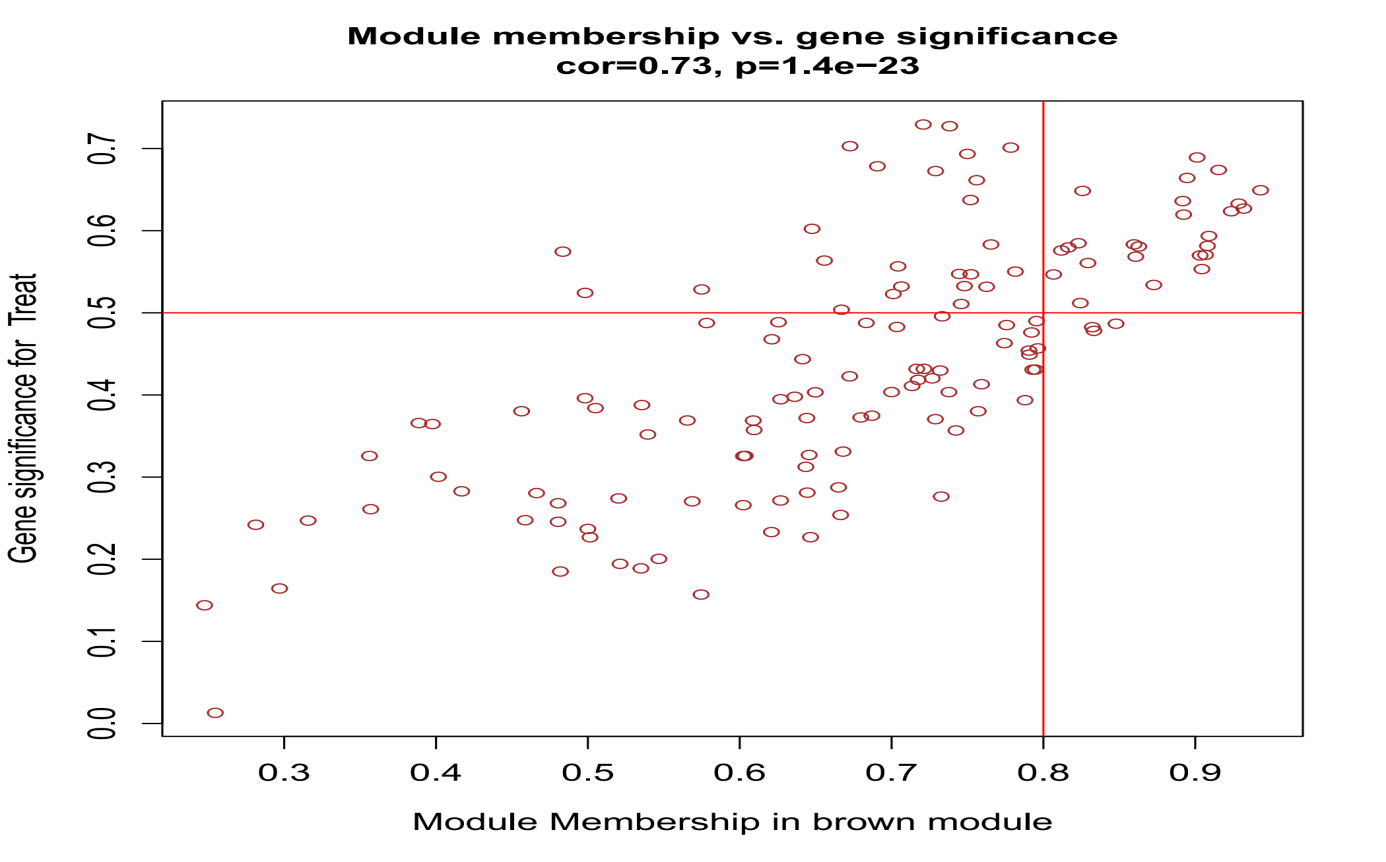
**

**Table S1**

**The primer sequences**

| **primer names** | | **sequence (5' to 3')** | **Length(nt)** |
| --- | --- | --- | --- |
| AKR1B1 (human）siRNA | Forward primer | AAGCCGTCTCCTGCTCAAC | 21 |
|  | Reverse primer | TGGTACACATGGGCACAGTC | 21 |
| CST6 (human）siRNA | Forward primer | TCCTGACGATGGAGATGGGG | 21 |
|  | Reverse primer | GTGAGGTCGACGTGGTCTCC | 21 |
| CTHRC1(human）siRNA | Forward primer | GATCCCCAAGGGGAAGCAAA | 21 |
|  | Reverse primer | TTTCAGGGCTTCCTTGGTCC | 21 |
